# Development of a Score to Predict the Paroxysmal Atrial Fibrillation in Stroke Patients: The Screening for Atrial Fibrillation Scale

**DOI:** 10.3389/fneur.2022.900582

**Published:** 2022-06-28

**Authors:** Laura Amaya Pascasio, Miguel Quesada López, Juan Manuel García-Torrecillas, Antonio Arjona-Padillo, Patricia Martínez Sánchez

**Affiliations:** ^1^Stroke Unit, Department of Neurology, Torrecárdenas University Hospital, Almería, Spain; ^2^Biomedical Research Unit, Hospital Universitario Torrecárdenas, Almería, Spain; ^3^Instituto de Investigación Biomédica Ibs. Granada, Granada, Spain; ^4^Centro de Investigación Biomédica en Red de Epidemiología y Salud Pública (CIBERESP), Madrid, Spain; ^5^Department of Emergency Medicine, Hospital Universitario Torrecárdenas, Almería, Spain

**Keywords:** atrial fibrillation, ischemic stroke, embolic stroke, embolic stroke of undetermined source, ESUS

## Abstract

**Background and Purpose:**

An individual selection of ischemic stroke patients at higher risk of atrial fibrillation (AF) might increase the diagnostic yield of prolonged cardiac monitoring and render it cost-effective.

**Methods:**

The clinical, laboratory, and brain/cardiac imaging characteristics of consecutive ischemic stroke patients without documented AF were recorded. All patients underwent at least 72 h of cardiac monitoring unless AF was diagnosed before, transthoracic echocardiogram, blood biomarkers, and intracranial vessels imaging. A predictive grading was developed by logistic regression analysis, the screening for atrial fibrillation scale (SAFE).

**Results:**

A total of 460 stroke patients were analyzed to develop the SAFE scale, a 7-items score (possible total score 0–10): age ≥ 65 years (2 points); history of chronic obstructive pulmonary disease or obstructive sleep apnea (1 point); thyroid disease (1 point); NT-proBNP ≥ 250 pg/ml (2 points); left atrial enlargement (2 points); cortical topography of stroke, including hemispheric or cerebellar cortex (1 point); and intracranial large vessel occlusion (1 point). A score = 5 identified patients with paroxysmal AF with a sensitivity of 83% and a specificity of 80%.

**Conclusion:**

Screening for atrial fibrillation scale (SAFE) is a novel and simple strategy for selecting ischemic stroke patients at higher risk of having AF who can benefit from a more thorough etiological evaluation. External validation of SAFE in a multicenter study, with a larger number of patients, is warranted.

## Introduction

Atrial fibrillation (AF) is the most common sustained arrhythmia and one of the most important causes of acute ischemic stroke (1). In many cases, AF is paroxysmal and a large proportion of patients remain undiagnosed (2). Recent clinical trials failed to prove the superiority of direct oral anticoagulation compared to standard antiplatelet treatment for secondary stroke prevention in unselected patients meeting the definition of embolic stroke of undetermined source (ESUS) (3–5), which supports the need for an accurate diagnosis.

The real prevalence of AF among stroke patients is unknown since only a small number of patients benefit from prolonged cardiac monitoring due to its costs and limited availability in daily clinical routine. Furthermore, recent evidence supports that the diagnosis of AF in patients with atherothrombotic or lacunar stroke is not negligible (6).

Several factors have been related to the presence of paroxysmal AF, including older age, female sex, previous history of hypertension, thyroid pathology, chronic obstructive pulmonary disease (COPD), heart failure or ischemic heart disease, higher National Institutes of Health Stroke Scale (NIHSS) score, left atrial enlargement, P-wave duration and morphology, brain natriuretic peptide, and HDL-cholesterol concentrations (7, 8). Other clinical factors like a history of obstructive sleep apnea (OSA) and hyperthyroidism have been associated with a greater AF recurrence after catheter ablation (9, 10).

In this way, individual risk evaluation for the presence of paroxysmal AF after an ischemic stroke may help to identify patients who can be targeted for more thorough evaluation strategies to assess the presence of occult AF.

The present study aims to determine the clinical, laboratory, brain-image, and echocardiographic features associated with the diagnosis of AF in ischemic stroke patients without prior history of AF. Second, to develop a score, the screening for atrial fibrillation scale (SAFE), to identify patients with an increased risk of occult AF.

## Materials and Methods

### Type of Study and Patient Selection

A retrospective analysis was performed with data from subjects who were registered in a prospectively collected stroke registry from consecutive patients admitted to the Neurology department of the Torrecárdenas University Hospital, Almería, Spain, during the first 72 h within symptoms onset, between January 2018 and May 2021. The study was approved by the local ethics committee and patient consent was not sought due to the retrospective nature of the study. The data that support the findings are available from the corresponding author on reasonable request.

#### Inclusion Criteria

- Subjects ≥ 18 years old with the diagnosis of ischemic stroke or transient ischemic attack.
- Cardiac monitoring ≥ 72 h (median time until AF diagnosis in a previous exploratory analysis carried out in our center) or shorter if an earlier AF diagnosis was performed.
- Determination of N-terminal prohormone of brain natriuretic peptide (NT-proBNP) within the first 48 h after admission for the index stroke.
- Evaluation of intracranial arteries by CT-angiography within the first 48 h after symptom's onset. A minority of patients who did not benefit from CT-angiography were included if brain MRI (time-of-flight sequences) was available.
- Hearth evaluation with transthoracic echocardiography (TTE) during or within 3 months after hospitalization.

#### Exclusion Criteria

- Known history of AF or AF diagnosis on the initial ECG.
- Presence of other major cardioembolic diseases, such as severe ventricular dysfunction (forced expiratory volume in 1 s <45%), mechanical valve prosthesis carrier, and rheumatic mitral valvulopathy.
- Incomplete etiological assessment, as previously described.

All patients were monitored during hospitalization either with telemetry or non-insertable Holter. The telemetry system consisted of a 3-lead ECG recorder with an automated arrhythmia detection function which is reviewed daily by the responsible neurologist. Additional monitoring with prolonged Holter was at the discretion of the neurologist in charge. The diagnosis of AF was made according to the 2020 ESC guidelines for the diagnosis and management of AF, a standard 12-lead ECG recording or a single-lead ECG tracing of ≥ 30 s showing heart rhythm with no discernible repeating P waves and irregular RR intervals (11). NT-proBNP was measured by a central laboratory on the Roche Elecsys assay (analytical range 10–35,000 pg/ml) and the echocardiographic measurements and calculations followed the recommendations of the American Society of Echocardiography (ASE) (12, 13). Left atrial enlargement was defined as a left atrial volume >34 ml/m^2^ or a left atrial area >20 cm^2^.

Demographic information and characteristics of the index stroke were retrieved. The presence of cardiovascular risk factors, any type of cardiopathy, thyroid disease, OSA, or COPD were registered. Thyroid disease was defined as the history of hyperthyroidism or hypothyroidism, with specific treatment or not at the moment of admission, and diagnosis of hyperthyroidism or hypothyroidism during hospitalization for the index stroke. Subclinical hypothyroidism or hyperthyroidism that did not require treatment was excluded. Likewise, information regarding echocardiography and brain imaging (either CT or MRI), intracranial arteries, laboratory tests, and continuous ECG monitoring was collected. Intracranial large vessel occlusion was defined as any identified occlusion in an intracranial artery, including intracranial carotid artery and vertebral artery (V4 segment) that was responsible for the patient's symptoms. Intracranial occlusions in the context of tandem lesions were excluded.

The patients with non-lacunar brain infarct without proximal arterial stenosis or cardioembolic sources, after TTE assessment and 24-h monitoring, were classified as ESUS, following the classical definition (3).

### Statistical Analysis

Data analysis was performed with the SPSS 27.0 program for macOS. Univariate analysis was performed with the *X*^2^ or Fisher's exact test to determine differences in the explored variables between patients with AF and non-AF. Continuous variables were analyzed with the *t*-test or the Mann–Whitney test when appropriate. Values of *p* <0.05 were considered significant. A threshold value was established for continuous variables (age, NT-proBNP) using a ROC (receiver-operating characteristic). Variables with a *p*-value <0.2 in the univariate analysis were included in a backward stepwise logistic regression multivariate analysis. To generate the scale scores, the Sullivan method (14) was applied, which takes into account each coefficient of the multivariate logistic regression model and divides them by the lowest of all. The score obtained is rounded to the nearest integer, this being the weight that each factor will have in the final score.

The ability to predict AF of the score was assessed with a ROC curve, for all the included patients and for the population meeting the ESUS criteria. The areas under the ROC curves (AUC) for the score were compared with those for the NT-proBNP and left atrial enlargement and with those for previously developed AF predictive scores. The model was internally validated by means of resampling or bootstrapping performing 1,000 iterations (15). An adequate validation was considered if the identified variables were supported by the model after the resampling procedure. Finally, the predictive potential of the new model was evaluated with a ROC curve.

## Results

**Figure 1:**
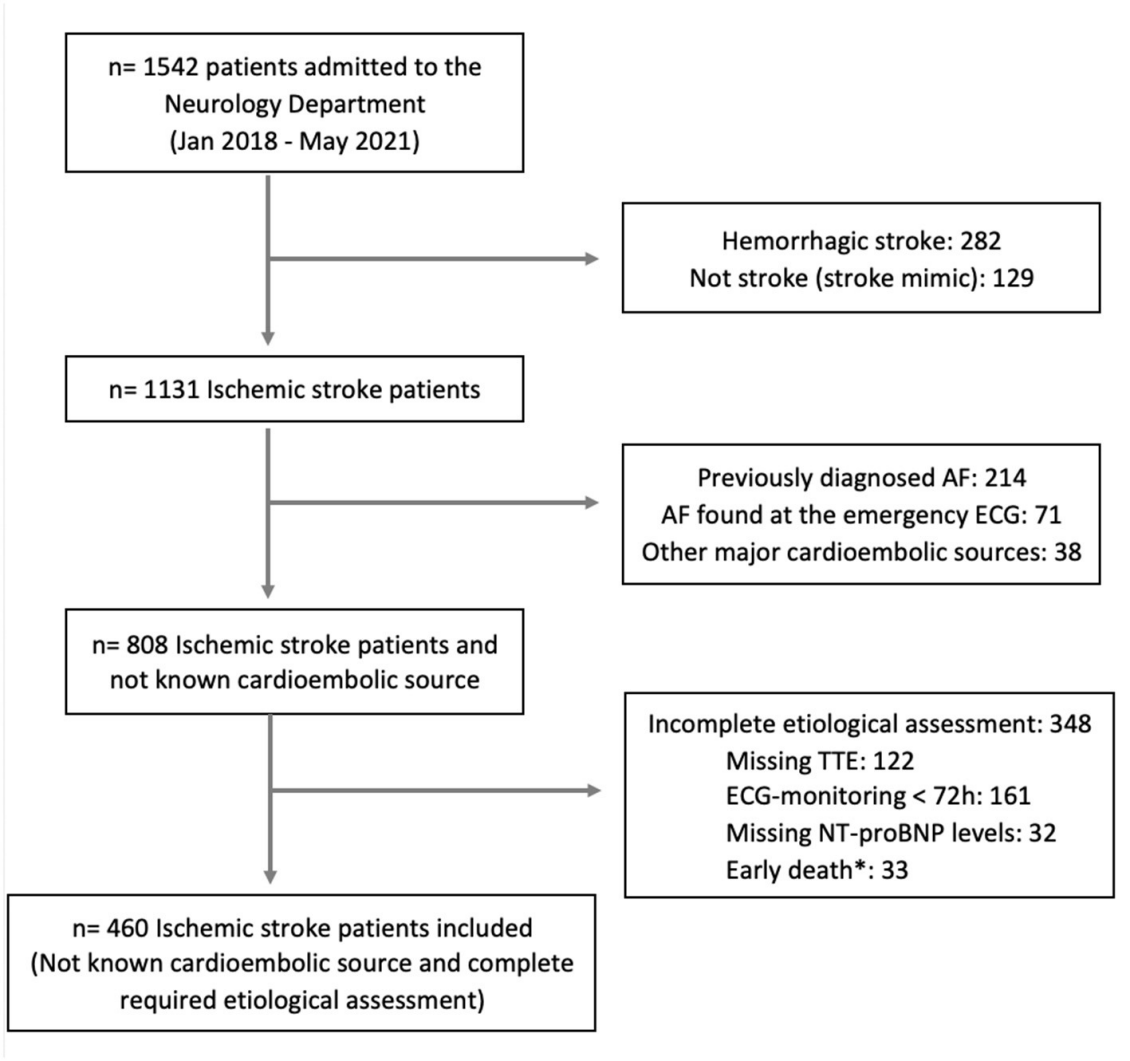
Study flowchart showing the patient selection process. *Incomplete assessment due to death within the first 72 h after admission, AF, atrial fibrillation; NT-proBNP, N-terminal prohormone of brain natriuretic peptide; TTE, transthoracic echocardiogram.

A total of 1,542 patients admitted to the Neurovascular Unit within the established period were assessed, of which 460 met the inclusion criteria and were selected for analysis. A flow diagram with the patient selection process is shown in Figure 1. The median age [interquartile range (IQR)] was 65 years (16) and 308 (67%) were men. Among them, 102 (22.2%) patients were diagnosed with AF within a maximum period of 12 months following the index stroke. The diagnosis was performed by in-hospital monitoring in 81 (79.4%) subjects and by 28-day monitoring in 18 (17.6%) subjects. The rest were found to have AF in routine visits. Distributions of cardiovascular risk factors, comorbidities, baseline NIH Stroke Scale (NIHSS) scores, and further patient characteristics are described in Table 1.

**Table 1:** Baseline characteristics and univariate analysis.

**Variable**	**All patients** **(*n* = 460)**	**Atrial fibrillation** ***n* = 102)**	**Non-atrial fibrillation** **(*n* = 358)**	***P-*value**
**Demographic data, vascular risk factors and comorbidities**				
Median age (IQR), years	65 (56–74)	74 (69–81)	62 (53–71)	**<0.001**
Age ≥ 65 years, *n* (%)	232 (50.4%)	88 (86.3%)	144 (40.2%)	**<0.001**
Male, *n* (%)	308 (67%)	53 (52%)	255 (71.2%)	**<0.001**
Arterial hypertension, *n* (%)	276 (60%)	76 (74.5%)	200 (55.9%)	**0.001**
Diabetes mellitus, *n* (%)	114 (24.8%)	22 (21.6%)	92 (25.7%)	0.394
Dyslipidemia, *n* (%)	191 (41.5%)	48 (47.1%)	143 (39.9%)	0.198
Smoking, *n* (%)	240 (52.2%)	40 (39.6%)	200 (55.9%)	**0.004**
Enolism, *n* (%)	37 (8%)	5 (5%)	32 (8.9%)	0.194
Ischemic heart disease, *n* (%)	33 (7.2%)	7 (6.9%)	26 (7.3%)	0.890
Chronic renal failure, *n* (%)	41 (8.9%)	14 (13.7%)	27 (7.6%)	0.054
COPD, *n* (%)	47 (10.2%)	22 (21.6%)	25 (7%)	**<0.001**
OSA, *n* (%)	39 (8.5%)	11 (10.8%)	28 (7.8%)	0.343
COPD or OSA	77 (16.7%)	30 (29.4%)	47 (13.1%)	**<0.001**
Thyroid disease, *n* (%)	33 (7.2%)	14 (13.7%)	19 (5.3%)	**0.004**
Previous stroke, *n* (%)	62 (13.5%)	20 (19.6%)	42 (11.7%)	**0.040**
**Laboratory features**				
NT-proBNP levels, median (IQR), pg/mL	186 (65–555)	784 (298–1632)	125 (49–319)	**<0.001**
NT-proBNP ≥ 250 pg/mL, n (%)	192 (41.7%)	81 (79.4%)	111 (31%)	**<0.001**
**Echocardiographic features**				
Left atrial enlargement, *n* (%)	126 (27.4%)	61 (59.8%)	65 (18.2%)	**<0.001**
Left ventricular hypertrophy, *n* (%)	181 (39.3%)	37 (36.3%)	144 (40.2%)	0.471
Diastolic dysfunction, *n* (%)	162 (36%)	34 (34.3%)	128 (36.5%)	0.697
Valvulopathy other than rheumatic mitral disease, *n* (%)	31 (6.7%)	11 (10.8%)	20 (5.6%)	0.065
**Neuroimaging features**				
Cortical topography of stroke, *n* (%)	276 (60%)	83 (81.4%)	193 (53.9%)	**<0.001**
Intracranial large vessel occlusion, n (%)	147 (32%)	52 (51%)	95 (26.5%)	**<0.001**
Chronic cortical stroke, *n* (%)	41 (8.9%)	18 (17.6%)	23 (6.4%)	**<0.001**
**Stroke characteristics**				
NIHSS score at admission, median (IQR)	4 (2–9)	7 (3–13)	3 (2–8)	**<0.001**
Transient ischemic attack, *n* (%)	15 (3.3%)	4 (3.9%)	11 (3.1%)	0.670
Stroke meeting ESUS criteria (24 h cardiac monitoring)	227 (49.3%)	81 (79.4%)	146 (40.8%)	**<0.001**
**Atrial fibrillation assessment**				
Telemetry duration, median (IQR), days	4 (3–6)	4 (2–5)	4 (3–6)	**0.019**
Time until diagnosis (telemetry), Median (IQR), days	2 (1–4)	2 (1–4)	N.A.	N.A.
24-h Holter monitoring, *n* (%)	79 (17.2%)	17 (16.7%)	62 (17.3%)	0.878
28-days Holter monitoring, *n* (%)	79 (17.2%)	19 (18.6%)	60 (16.8%)	0.659

*COPD, chronic obstructive pulmonary disease; ESUS, Embolic stroke of undetermined source; N.A., Not applicable; NT-proBNP, N-terminal prohormone of brain natriuretic peptide; OSA, obstructive sleep apnea*.

Patients diagnosed with paroxysmal AF had a higher median age [74 (IQR 69–81) vs. 62 (IQR 53–71), *p* <0.001] and more often experienced arterial hypertension (74.5 vs. 55.9%, *p* 0.001), thyroid dysfunction (13.7 vs. 5.3%, *p* 0.004), and COPD (21.6 vs. 7%, *p* <0.001). In addition, they were less frequently current or former smokers (39.6 vs. 55.9%, *p* 0.004). Stroke severity measured by the NIHSS was significantly higher in patients with AF [7 (IQR 3–13) vs. 3 (IQR 2–8), *p* <0.001] and they presented more frequently with intracranial large vessel occlusion (51 vs. 26.5%, *p* <0.001) and an infarction affecting the cortex (81.4 vs. 53.9%, *p* <0.001). The proportion of patients with left atrial enlargement was significantly higher in the AF group (59.8 vs. 18.2%), and the median NT-proBNP levels [784 (IQR 298–1632) vs. 125 (IQR 49–319), *p* <0.001].

For the development of the proposed risk score, logistic regression multivariate analysis was performed and is detailed in Supplementary Table 1 of the Supplementary Material. The final multivariate model included seven covariates: age ≥ 65 years [OR: 4.2 (95% CI, 2.1–8.3)]; history of COPD or OSA [OR: 2.4 (95% CI, 1.2 4.6)]; thyroid disease, either hypothyroidism or hyperthyroidism [OR: 2.8 (95% CI, 1.1–7.3)]; NT-proBNP ≥ 250 pg/ml [OR: 4.0 (95% CI, 2.2–7.5)]; left atrial enlargement [OR: 3.6 (95% CI, 2.1–6.4)], cortical topography of stroke, including hemispheric or cerebellar cortex [OR: 2.2 (95% CI, 1.1–4.2)]; and intracranial large vessel occlusion [OR: 2.0 (95% CI, 1.1–3.6)].

**Figure 2:**
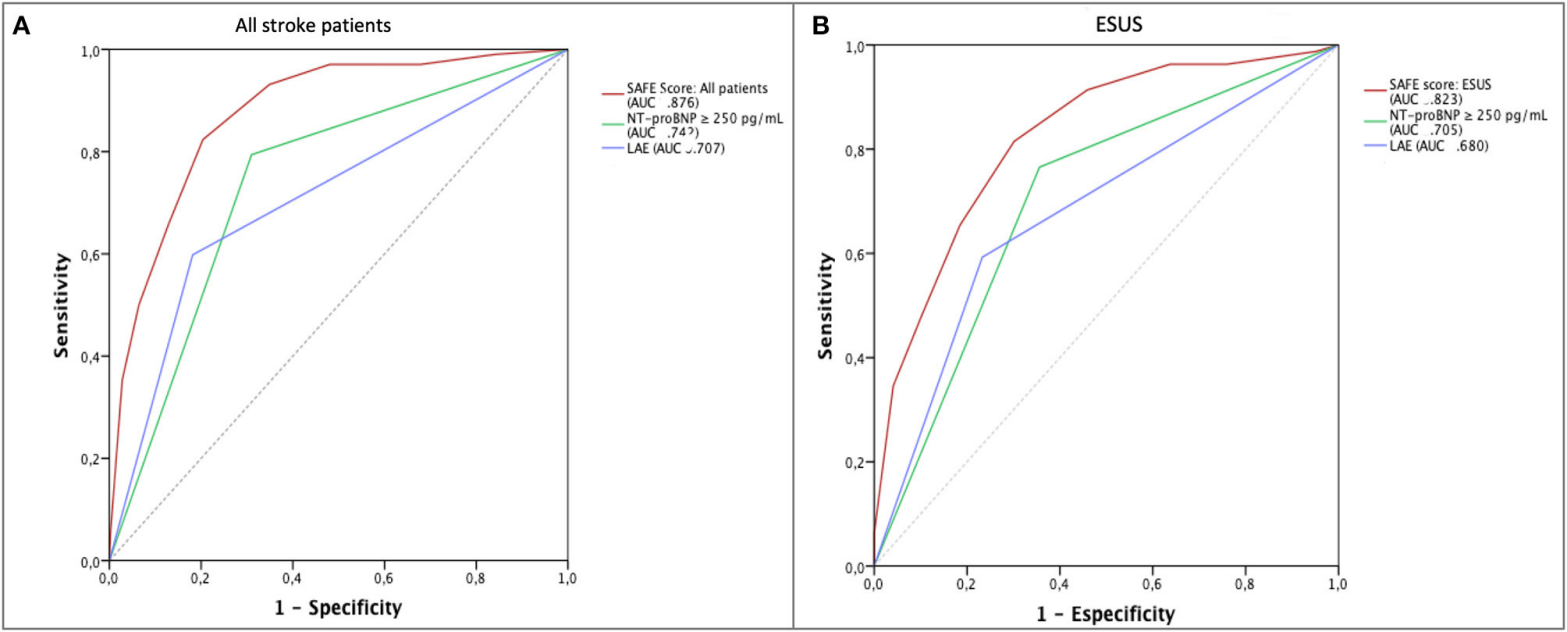
AF prediction by SAFE. **(A)** Prediction of new-onset AF by the SAFE scores (AUC 0.876), compared to NT-proBNP levels (AUC 0.742) and left atrial enlargement (AUC 0.707) for all stroke-patients, *p* <0.05. **(B)** Prediction of new-onset AF by the SAFE scores (AUC 0.823), compared to NT-proBNP levels (AUC 0.705) and left atrial enlargement (AUC 0.680) for stroke-patients meeting the ESUS criteria after 24 h cardiac monitoring, *p* <0.05. AF, atrial fibrillation; AUC, area under the curve; ESUS, Embolic stroke of undetermined source; LAE, left atrial enlargement; NT-proBNP, N-terminal prohormone of brain natriuretic peptide; SAFE, screening for atrial fibrillation scale.

**Figure 3:**
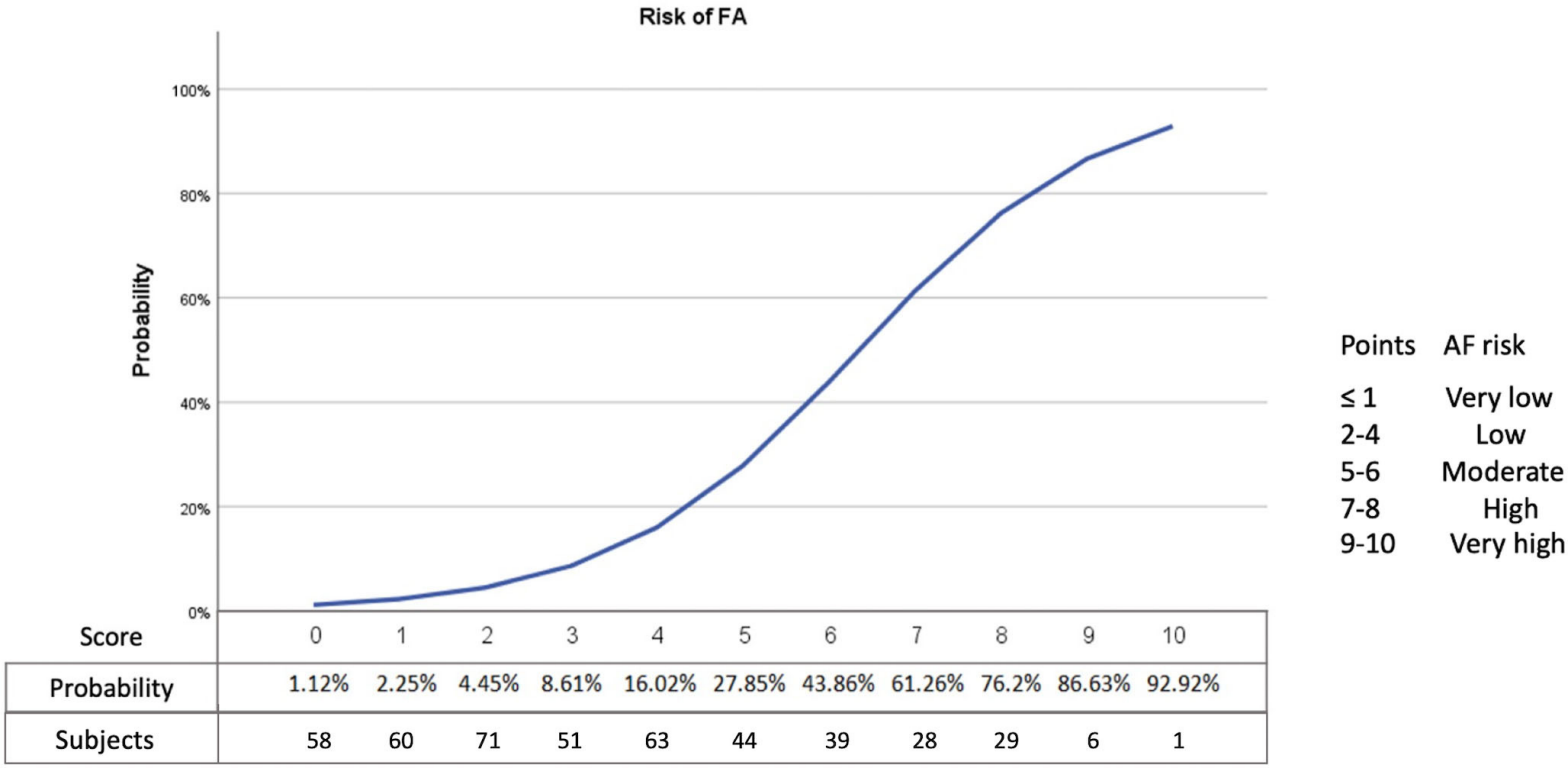
Representation of the risk of AF (percentage) according to the SAFE score. AF, atrial fibrillation.

**Figure 4:**
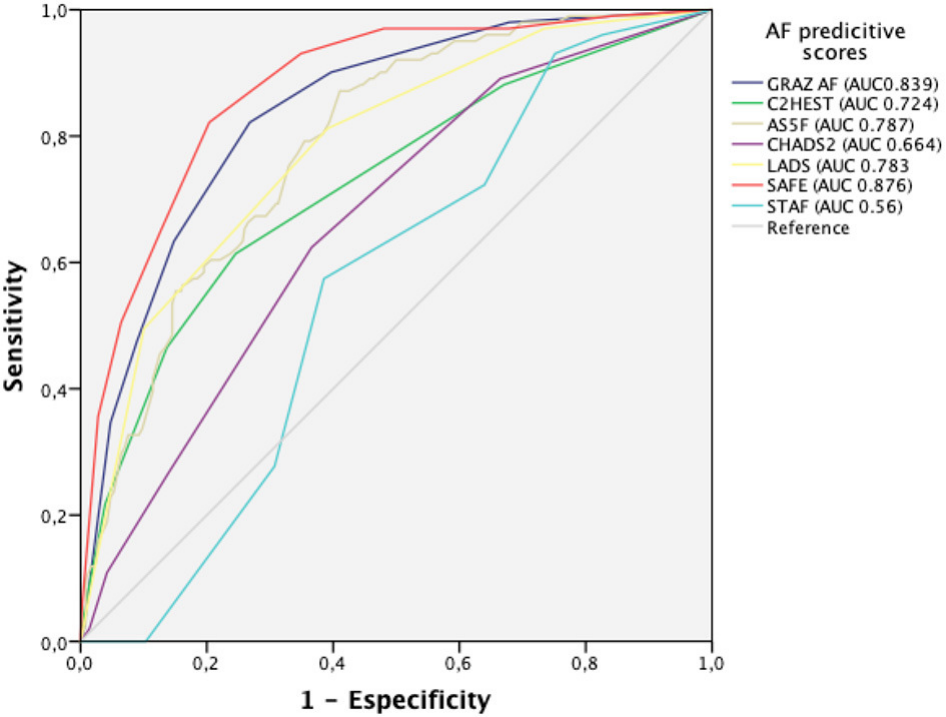
Prediction of atrial fibrillation by SAFE compared to other predictive scores. SAFE showed an AUC of 0.876 (95% CI 0.838–0.913, *p* <0.001). The AUC of GRAZ AF was 0.839 (95% CI 0.797–0.881); AUC of C2HEST 0.724 (95% CI 0.665–0.783), AUC of AS5F was 0.787 (95% CI 0.741–0.834), for CHADS2, the AUC was 0.664 (95% CI 0.607–0.7222); LADS had an AUC of 0.783 (95% CI 0.733–0.832) and STAF 0.56 (95% IC 0.504–0.616). AUC, area under the curve.

The score derived from logistic regression analysis, following the Sullivan method (14), is presented in Table 2. The area under the curve of the score values obtained for later detection of paroxysmal AF was 0.88 (95% CI 0.84–0.91). SAFE was found to be superior for AF prediction to blood biomarkers (NT-proBNP levels) and echocardiographic features (left atrial enlargement) alone, both in all-stroke patients and specifically in the ESUS population, as shown in Figure 2. A score ≥ 5 identified patients with paroxysmal AF with a sensitivity of 83%, a specificity of 80%, and a negative predicted value of 94%. A total score of 7 was associated with a 61% risk of occult AF (Figure 3). The AUC of SAFE was compared to the ability to predict AF of the previously described score. SAFE showed a greater AUC compared to other scores in the present cohort as is represented in Figure 4. The main characteristics of the previously developed scores, compared to SAFE, are presented in Table 3. The internal validation of the model supported the variables previously identified and is presented in the Supplemental Material.

**Table 2:** Proposed scoring in SAFE.

**Variable**	**Points**
**Age**
<65 years	0
≥65 years	2
COPD or OSA*	1
Thyroid disease*	1
**NT-proBNP**
<250 pg/mL	0
≥250 pg/mL	2
Left atrial enlargement**	2
Cortical topography of stroke	1
Intracranial large vessel occlusion	1
Total	0 to 10

*Previous history or newly diagnosed; **All measurements followed the ASE recommendations (12). COPD, chronic obstructive pulmonary disease; NT-proBNP, N-terminal prohormone of brain natriuretic peptide; OSA, obstructive sleep apnea.

**Table 3:** Characteristics of previously developed AF predictive scales and the SAFE score.

**Characteristics**	**STAF (17)**	**LADS (18)**	**iPAB (19)**	**CHADS2 (20)**	**HAVOC (21)**	**AS5F (22)**	**C2HEST (16)**	**GRAZ AF (23)**	**SAFE**
Population	*N* = 456 Previous AF included	*N* = 953 Previous AF included	*N* = 431 Previous AF excluded	*N* = 57636 Previous AF excluded	*N* = 9589 Previous AF excluded	*N* = 1135 Previous AF excluded	*N* = 240459	*N* = 150 Previous AF excluded	*N* = 460 Previous AF excluded
Variables in the Score	Age, NIHSS, LAE, Vascular ethiology*	LAE, Age, Stroke vs. TIA, No Smoking	Previous arrhythmia^∧^, LAE, BNP	Congestive heart failure, Age, Hypertension, Diabetes Mellitus, Prior stroke/TIA	Hypertension, Age, Non-embolic Valve disease, Peripheral Vascular disease, Obesity, Heart failure, Coronary disease	Age, NIHSS	Coronary disease, COPD, Hypertension, Age, Systolic heart failure, Thyroid disease	Age, Recurrent stroke/multi-territory brain infarct, Prior cortical/cerebellar infarction, Echocardiography: Left ventricular EF/LAE, ECG/Monitoring, NT-proBNP (≥505 pg/ml)	Age, LAE, COPD/OSA, NT-proBNP (≥250 pg/mL), Cortical topography of stroke, intracranial large vessel occlusion
Indicators	AUC 0.94 (95% CI 0.92–0.96). *Score ≥5:* Sensitivity 89% Specificity 88%	AUC not reported Score ≥4 Sensitivity 85.5% Specificity 53.1% PPV 24.7% NPV 95.3%	AUC 0.90 (95% CI 0.85–0.94). *Score ≥ 2:* Sensitivity 93% Specificity 71%	AUC not reported *Score ≤ 3:* Sensitivity 42.5% Specificity 77.6% PPV 30.4% NPV 85.4%	AUC 0.77 (95% CI not reported)	AUC 0.78 (95% CI not reported)	AUC 0.734 (95% CI 0.732–0.736)	AUC 0.85 (95% CI 0.78–0.92) *Score ≥4:* Sensitivity 92% Specificity 67% NPV 98%	AUC 0.88 (95% CI 0.84–0.91). *Score ≥ 5:* Sensitivity 83% Specificity 80% NPV 94% PPV 53%

*Defined by the absence of symptomatic extra- or intracranial stenosis ≥ 50%, symptomatic arterial dissection, clinic-radiological lacunar syndrome, ^∧^any type of arrhythmia except atrial fibrillation. AF, atrial fibrillation; AUC, area under the curve; BNP, brain natriuretic peptide; CI, confident interval; COPD, chronic obstructive pulmonary disease; LAE, left atrial enlargement; NPV, negative predictive value; NT-proBNP, N-terminal prohormone of brain natriuretic peptide; OSA, obstructive sleep apnea; PPV, positive predictive value; TIA, transient ischemic attack.

## Discussion

The present study proposes a simple and novel score to identify ischemic stroke patients at higher risk of having paroxysmal AF as the main cause of the stroke. SAFE combines clinical, echocardiographic, neuroimaging features, and blood biomarkers and has been developed in an ischemic stroke cohort in which 49% met the definition criteria for ESUS.

Detecting occult AF in patients who have suffered an ischemic stroke is currently a challenge. In patients with cryptogenic cerebral infarction, prolonged ECG monitoring with external or insertable loop recorders has detected occult AF in 15–30% of cases (24, 25). The major limitations of these devices are their restricted availability and elevated cost. In this context, several risk scales have been developed before to select the best candidates to undergo prolonged monitoring. However, most of them focus on clinical or echocardiographic characteristics, not including neuroimaging or blood biomarkers (16–18, 20–22, 26–28). Three previous predictive scores included blood biomarkers, BNT in two cases (19, 29), and NT-proBNP in the most recently published one (23). However, TTE was not available in all cases and patients were only monitored for 24 h in one of them (29) and the second, the iPAB score, did not include well-established clinical risk factors for AF, such as age (19). The Graz AF risk score (23) included NT-proBNP levels as a covariate in the risk model and was developed in a cohort of cryptogenic ischemic strokes that had undergone in-hospital cardiac monitoring and an additional 24 h Holter. It was limited by the sample size, with only 24 patients diagnosed with AF during the follow-up period and very few patients benefiting from prolonged heart monitoring (23). Furthermore, the suggested NT-proBNP cut-off value was 505 pg/ml, higher than proposed in previous studies assessing the risk of AF in patients with stroke (30, 31). In the present cohort, the proposed cut-off value was 250 pg/ml, with an 80% sensitivity and 70% specificity in the ROC curve and in line with the results that have been published previously (30, 31). We hypothesize that in patients with occult AF, NT-proBNP levels might remain relatively low compared to patients with a high AF burden.

The strengths of SAFE, compared to previously developed AF risk scores, are first, the homogeneous etiological evaluation of all the included subjects. Both, the AF and the non-AF groups benefited from similar monitoring times, with a minimum 72-h period if AF was not found before. Likewise, the same percentage of patients received prolonged cardiac monitoring in both groups, which makes the etiological work-up comparable. Second, the included covariates in the predictive model included not only clinical, cardiac, or blood biomarkers but also a combination of all of them, with a greater prediction power than each of them individually. In the same way, SAFE has proven its predictive potential specifically in the ESUS population, representing a significant proportion of the included subjects. The best cutoff level of 5 points demonstrated high sensitivity and specificity, 83 and 80%, respectively, for detecting paroxysmal AF in ischemic stroke patients. In our cohort, only 5% of patients with SAFE scores <5 were diagnosed with AF during the follow-up period, which results in a high negative predictive value of 94%. Consequently, such patients are very unlikely to have occult AF as a cause of the stroke, and other possible stroke etiologies should be ruled out.

There are several limitations to this study, such as its retrospective nature, a possible patient selection bias attributable to monocentric recruitment, and to the heterogeneity of the etiological approach, at the discretion of the neurologist in charge. Other limitations could be the lack of prolonged monitoring for every included patient and the absence of ECG variables, such as p-wave duration or morphology. However, these variables could complicate the implementation of the scale and limit the generalization of its use.

## Conclusion

Screening for atrial fibrillation scale (SAFE) is a novel and simple strategy for selecting ischemic stroke patients at higher risk of having AF who can benefit from a more thorough etiological evaluation. External validation of SAFE in a multicenter study, with a larger number of patients, is warranted.

## Data Availability Statement

The original contributions presented in the study are included in the article/Supplementary Material, further inquiries can be directed to the corresponding author.

## Ethics Statement

The studies involving human participants were reviewed and approved by Ethics Committee of Almeria. Written informed consent for participation was not required for this study in accordance with the national legislation and the institutional requirements.

## Author Contributions

LA, PM, and JG-T conceived and designed the methodology of the study. LA and MQ analyzed the collected data and drafted the manuscript. All authors were involved in the evaluation and interpretation of the study results and review and approval of the manuscript.

## Funding

The investigators were granted funds by the Carlos III Institute of Health (RICORS-ICTUS and RD21/0006/0010).

## Conflict of Interest

The authors declare that the research was conducted in the absence of any commercial or financial relationships that could be construed as a potential conflict of interest.

## Publisher's Note

All claims expressed in this article are solely those of the authors and do not necessarily represent those of their affiliated organizations, or those of the publisher, the editors and the reviewers. Any product that may be evaluated in this article, or claim that may be made by its manufacturer, is not guaranteed or endorsed by the publisher.
