# Supplementary material for: Development of a Score to Predict the Paroxysmal Atrial Fibrillation in Stroke Patients: The Screening for Atrial Fibrillation Scale
Source: Front Neurol. 2022 Jun 28;13:900582. doi: 10.3389/fneur.2022.900582 (PMC9274122; doi:10.3389/fneur.2022.900582)
Supplement: Supplementary file 1 [file Data_Sheet_1.docx]

**SUPPLEMENTAL MATERIAL**

**Table S1**. Logistic regression multivariate analysis.

| Variable | B | SE | Wald | *p* | OR | CI 95% IC |
| --- | --- | --- | --- | --- | --- | --- |
| Age ≥ 65 | 1.439 | 0.345 | 17.373 | .000 | 4.217 | 2.143 – 8.295 |
| Intracranial large vessel occlusion | 0.700 | 0.295 | 5.634 | .018 | 2.013 | 1.130 – 3.588 |
| Cortical topography of stroke | 0.788 | 0.334 | 5.559 | .018 | 2.200 | 1.142 – 4.237 |
| Left atrial enlargement | 1.293 | 0.286 | 20.412 | .000 | 3.643 | 2.079 – 6.382 |
| COPD or OSA | 0.865 | 0.342 | 6.375 | .012 | 2.374 | 1.213 – 4.644 |
| Thyroid disease | 1.031 | 0.487 | 4.489 | .034 | 2.804 | 1.080 – 7.278 |
| NT-ProBNP ≥ 250pg/mL | 1.397 | 0.313 | 19.966 | .000 | 4.043 | 2.191 – 7.462 |

CI: Confident interval; COPD: chronic obstructive pulmonary disease; NT-proBNP: N-terminal prohormone of brain natriuretic peptide; OSA, obstructive sleep apnea. SE: Standard Error.

**Table S2.** Probability of paroxysmal AF and risk category according to the SAFE punctuation.

| Score | Probability of AF (95 IC) | Risk category |
| --- | --- | --- |
| ≤1 | 1.7% (1.6% - 1.8%) | Very low |
| 2-3 | 6.2% (5.8% - 6.6%) | Low |
| 4-5 | 20.9% (19.8% - 22%) | Moderate |
| 6-7 | 51.1% (49% - 53.2%) | High |
| >7 | 77.9% (76.7% - 79.2%) | Very high |

**Internal validation of SAFE**

*Bootstrapping*

The robustness of the model was tested by means of resampling or Bootstrapping. A number of 1.000 repeated samples were used. The variables previously identified in the model were maintained.

**Table S3**. Internal validation of the model by means of Bootstrapping.

| Variable | B | Bias | SE | *p* | 95% CI |
| --- | --- | --- | --- | --- | --- |
| Age ≥ 65 | 1.439 | 0.055 | 0.386 | .001 | 0.780 – 2.257 |
| Intracranial large vessel occlusion | 0.700 | 0.022 | 0.295 | .009 | 0.174 – 1.318 |
| Cortical topography of stroke | 0.788 | 0.014 | 0.344 | .019 | 0.149 – 1.530 |
| Left atrial enlargement | 1.293 | 0.035 | 0.311 | .001 | 0.721 – 1.926 |
| COPD or OSA | 0.865 | 0.015 | 0.374 | .010 | 0.145 – 1.577 |
| Thyroid disease | 1.031 | 0.021 | 0.398 | .005 | 0.255 – 1.867 |
| NT-ProBNP ≥ 250pg/mL | 1.397 | 0.041 | 0.327 | .001 | 0.830 – 2.119 |

CI: Confident interval; COPD: chronic obstructive pulmonary disease; NT-proBNP: N-terminal prohormone of brain natriuretic peptide; OSA, obstructive sleep apnea. SE: Standard Error.
